# Investigation of Volatiles Emitted from Freshly Cut Onions (*Allium cepa* L.) by Real Time Proton-Transfer Reaction-Mass Spectrometry (PTR-MS)

**DOI:** 10.3390/s121216060

**Published:** 2012-11-22

**Authors:** Mette Marie Løkke, Merete Edelenbos, Erik Larsen, Anders Feilberg

**Affiliations:** 1 Department of Engineering, Aarhus University, Blichers Allé 20, P.O. Box 50, Tjele DK-8830, Denmark; E-Mail: MetteM.Loekke@agrsci.dk; 2 Department of Food Science, Aarhus University, Kirstinebjergvej 10, Aarslev DK-5792, Denmark; E-Mails: Merete.Edelenbos@agrsci.dk (M.E.); Erik.Larsen@agrsci.dk (E.L.)

**Keywords:** PTR-MS, real time monitoring, volatile organic compounds, *Allium cepa* L

## Abstract

Volatile organic compounds (VOCs) in cut onions (*Allium cepa* L.) were continuously measured by PTR-MS during the first 120 min after cutting. The headspace composition changed rapidly due to the very reactive volatile sulfurous compounds emitted from onion tissue after cell disruption. Mass spectral signals corresponding to propanethial S-oxide (the lachrymatory factor) and breakdown products of this compound dominated 0–10 min after cutting. Subsequently, propanethiol and dipropyl disulfide predominantly appeared, together with traces of thiosulfinates. The concentrations of these compounds reached a maximum at 60 min after cutting. Propanethiol was present in highest concentrations and had an odor activity value 20 times higher than dipropyl disulfide. Thus, propanethiol is suggested to be the main source of the characteristic onion odor. Monitoring the rapid changes of VOCs in the headspace of cut onion necessitates a high time resolution, and PTR-MS is demonstrated to be a very suitable method for monitoring the headspace of freshly cut onions directly after cutting without extraction or pre-concentration.

## 1. Introduction

Different species of *Allium* are used worldwide as a common food or food ingredient. Freshly cut onion (*Allium cepa* L.) immediately brings tears to our eyes, and during cutting, a distinct onion odor develops. The tears and the odor are caused by very volatile and reactive sulfur compounds released after rupture of the onion cell structure.

**Figure 1.**
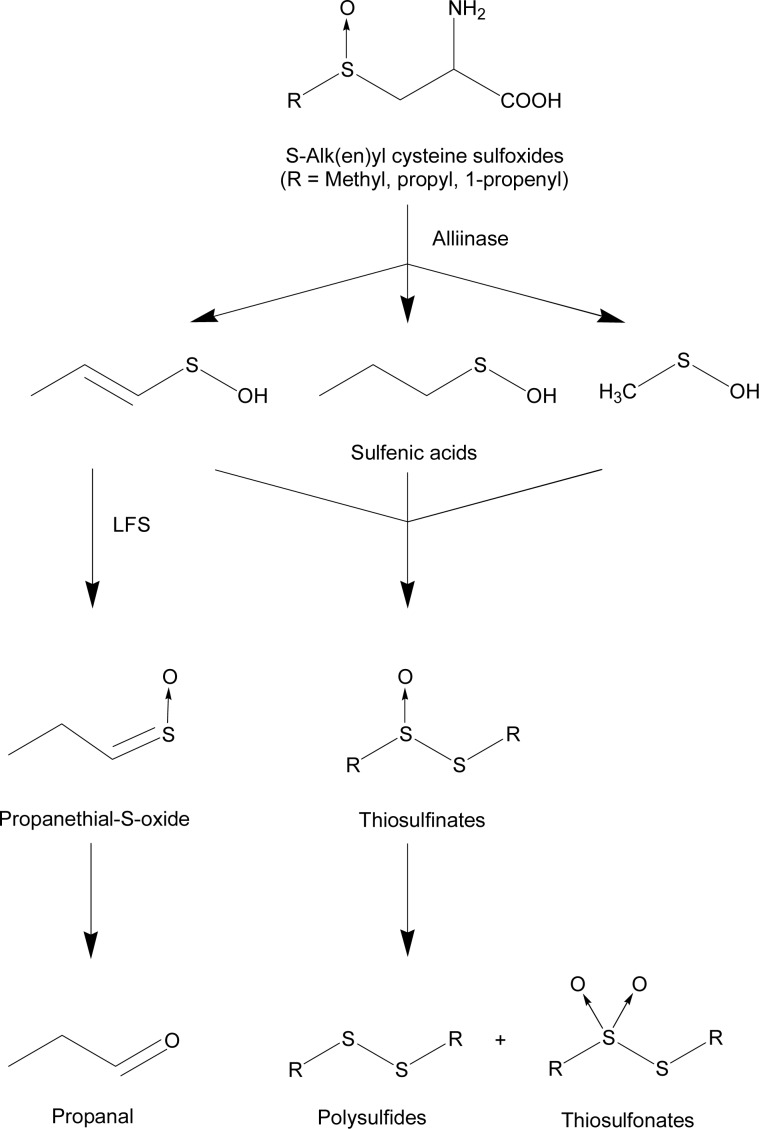
*Allium cepa* chemistry occurring after cutting [1–3]. LFS: lachrymatory factor synthase.

When onions are cut and the tissue is damaged, chemical reactions occurs giving rise to the characteristic sulfurous note of the onion odor [1–3]. Different sulfur compounds are formed when species of *Allium* are analyzed, as shown in Figure 1.

Upon rupture of the cell wall the alliinase enzyme is mixed with the aroma precursors; the cysteine sulfoxides, where the substituent is a methyl- ethyl-, propyl-, 1-propenyl-, or 2-propenyl-group (trivially named methiin, ethiin, propiin, isoalliin, and alliin, respectively). The structure and content of the sulfoxides is responsible for the flavor and odor of different *Allium* species like onion, garlic and leek [4–6]. The products of the enzymatic reaction between alliinase and cysteine sulfoxides are pyruvate, ammonia and various sulfenic acids depending on the S-attached substituents present in the cysteine sulfoxide. In onion the major sulfoxide is 1-propenyl cysteine sulfoxide [7,8]. The 1-propenyl sulfenic acid is primarily transformed into propanethial S-oxide, the onion lachrymatory factor (LF) [9], by LF synthase (LFS) [10]. LF seems to be present immediately after cutting with almost complete disappearance after 30 min [11,12]. In the presence of water, LF is degraded into propanal [3,8,13]. The other sulfenic acids condense into thiosulfinates, which are suggested to be responsible for the odor of freshly cut onions [1,11]. Thiosulfinates are relatively unstable and rearrange into polysulfides, thiosulfonates and other compounds [1,7,8].

The time-aspect is very important for the analysis of the volatile profile emitted from freshly cut onions. Immediately after cutting, LF is the major compound, but after 30 min it may no longer be detected, while other compounds are formed [7,12]. LF can easily be trapped by solid-phase microextraction (SPME) and measured by gas chromatography (GC) [8,11–13]. It seems that the LF is stable during GC analysis. In contrast, thiosulfinates easily degrade during trapping and GC-analysis, however, they are trapped in minor amounts by cold-trapping followed by high performance liquid chromatography coupled to mass spectrometry (HPLC-MS) [13] or by direct injection of a diethyl ether extract on a GC-MS [8,11]. Recently, Kubec *et al.*[14] used real time-mass spectrometry (DART-MS) for direct analysis of volatile organic compounds (VOCs) emitted from different species of *Allium*. In this experiment, a capillary was used to puncture the plant material, and the capillary was then positioned in the sampling position. The measurement started 1–2 s after cell rupture, and by this method mainly LF was detected, along with small amounts of thiosulfinates. When SPME or thermal desorption tubes are used for volatile analysis only LF and polysulfides are detected [3,7,8,11–13], supposedly due to degradation of thiosulfinates into polysulfides during sampling and GC analysis [11,14,15].

Consequently, the dynamics in the emission of volatiles from freshly cut onions call for a continuous analysis method with a simple sampling method that does not imply a potentially degrading pre-concentration step. Proton-transfer reaction-mass spectrometry (PTR-MS) is a relatively new technique for trace gas analysis of VOCs [16]. The method is based on chemical ionization by protonated water (H_3_O^+^) and has high selectivity and sensitivity and a fast response time [16–18]. Gas from the headspace is continuously drawn directly into the drift tube of the instrument, where volatiles with a proton affinity higher than water are ionized, and the ions are hereafter led to the detector. PTR-MS has been used for detection of volatiles from fruits and vegetables *i.e.*, apples [17,19], berry fruit [20–22] and broccoli [23]. The advantage of real-time monitoring and high time resolution makes PTR-MS an interesting choice for measuring VOCs emitted from freshly cut onion.

The aim of this research was to explore the applicability of using PTR-MS to determine volatile sulfur compounds emitted from freshly cut onions during the first 120 min after cutting. The dynamic PTR-MS results were compared with discrete detection on a GC equipped with a sulfur specific detection (GC-SCD) as a reference method. Odor Threshold Values (OTVs) were used to evaluate the impact of the analyzed volatile sulfur compounds for onion odor.

## 2. Experimental Section

### 2.1. Chemicals

All chemicals and authentic reference compounds were obtained from Aldrich or Fluka (Sigma-Aldrich A/S, Brøndby, Denmark) and were of analytical grade. The reference compounds hydrogen sulfide and methanethiol were gaseous standards at 5.06 ppm and 5.20 ppm, respectively, diluted in nitrogen (BOC, Surrey, UK). Dimethyl, dipropyl and diallyl thiosulfinate were synthesized by oxidation of the corresponding disulfide with peracetic acid according to the method of Moore and O’Connor [24]. The thiosulfinates were stored at −80 °C until analysis 6 days later.

### 2.2. Experimental Setup

**Figure 2.**
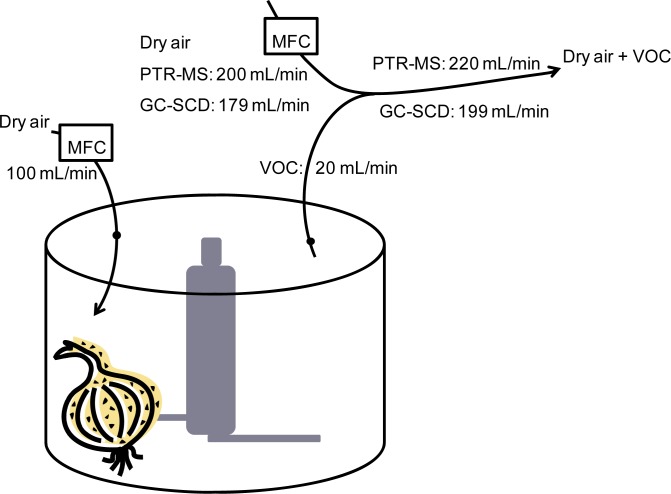
Experimental setup of sampling for PTR-MS or GC-SCD.

In order to detect the volatiles immediately after cutting of an onion a food processor (Kenwood FP250 multipro compact food processor, 2.1 L, 750W; Kenwood, Hampshire, UK) was modified and used as a combined cutter and sample compartment for the headspace analysis (Figure 2). Two holes was introduced in the lid and connected to Teflon tubes for inlet and outlet flow. The build in hole for insertion of material to the compartment was closed by duct tape, and besides this no attempts were done to make the sample compartment airtight. Instead the inlet flow was much higher than the outlet flow ensuring a minor positive pressure in the sample compartment, thus, potential leakages were acceptable. The inlet flow of dry zero-air (produced from pressurized air by using a cold trap with obtained dew point: ∼−30 °C and a charcoal filter) into the sample compartment was set to 100 mL/min. The outlet flow was diluted with dry zero-air at a ratio of 1:11. The dilution factor was controlled by the flow rate of the dry zero-air (200 mL/min for PTR-MS and 179 mL/min for GC-SCD) and the flow rate drawn by the instrument (220 mL/min for PTR-MS) or by a pump through the sample loop (199 mL/min for GC-SCD) (Figure 2). The flow rates of dry zero-air were controlled by mass flow controllers (El-flow, Bronkhorst, Ruurlo, The Netherlands). All connections were made with Teflon tubes and the measurements were performed at room temperature.

One yellow onion (*Allium cepa* L.) for each experiment was peeled and carefully trimmed by removing the outer layers to a size of 50–70 g in order to ensure that the onion fitted into the sample container. The trimming was performed 5–10 min before the onset of the VOC sampling. Background measurements were performed. Then an onion was introduced into the sample compartment, and the lid was closed. The cutting was performed at maximum speed for 10 s and the emitted volatiles were analyzed for up to 120 min after cutting. The sizes of the onion pieces were 2–10 mm. The measurements on PTR-MS and GC-SCD were done in triplicates using one onion for each experiment.

### 2.3. PTR-MS

#### 2.3.1. Settings

A PTR-MS instrument (HS-PTRMS, IONICON Analytic Gmbh, Innsbruck, Austria) was used at the following drift tube conditions: pressure of 2.1–2.2 mbar, temperature of 60 °C, and voltage at 600 V, corresponding to an E/N-value of ∼137 Td. Mass spectrometric data were collected over a mass range of *m/z* 21–200 using a dwell time of 0.2 s. The PTR-MS was setup for continuous acquisition of mass scans, and the measurement interval was ∼36 s. A mass dependent transmission curve based on a standard mixture of VOCs in concentrations close to 100 ppb (±10%; Restek, P/N 344423-PI) was used as a part of the quantification procedures.

The limit of detection (LOD, calculated as three times the standard deviation on blank samples) of our PTR-MS system with respect to volatile organic sulfur compounds is typically in the range of 0.02–0.15 ppb [25–27], which is lower than LOD of some alternative methods, e.g., GC-PFPD (pulsed flame photometric detector) (0.5–2.4 ppb, three times the standard deviation) [28] and GC-ASD (amperometric sulfur detector) (around 5 ppb) [29]. However, the LOD of the PTR-MS used in this study is higher than found for GC-ToF-MS (0.122 ppt, three times the standard deviation of the background noise) [30], and the method detection limit (MDL) is often expected to be much higher than the LOD [30].

#### 2.3.2. Identification and Quantification of VOCs

Identification of VOCs was based on commercially available or synthesized reference compounds. Mass spectra for identification were obtained by adding a few drops of reference compounds to a 2 mL glass vial closed with a polypropylene cap with PTFE/silicone septa (Varian, Palo Alto, CA, USA). A 1 mm hole was introduced into the septum with a medical needle. This vial was placed in a 100 mL blue cap flask with inlet and outlet tubing for PTR-MS measurements. To calculate concentrations in Section 3.5, the proton transfer reaction rate coefficients reported in Table 1 were used. For all other Figures, a coefficient of 2 × 10^−9^ cm^3^/s was used to provide concentration levels [16]. The reference spectra were used to calculate the relation between the protonated parent ion and all the fragments in the reference spectra, e.g., for propanal: [*m/z* 59]/calibration factor. The concentration of hydrogen sulfide shown in Section 3.5 below was determined based on a humidity-dependent calibration as described in [25,26]. Before data analysis, a background spectrum was subtracted.

#### 2.3.3. Investigation of Fragmentation Pattern at Different E/N-Values

In order to investigate the fragmentation pattern during the first 20 min after cutting, the E/N number was changed by changing the voltage in separate experiments. The investigated voltages were 600, 550, 500, 480, 460, and 440 V giving E/N-values of approximately 137, 127, 115, 111, 106, and 102 Td, respectively. Separate experiments with onions were conducted for each E/N number. The voltage was changed to the set-value and background scans were measured. Then an onion was introduced to the sample compartment and cut as described in Section 2.2. Hereafter, the volatile profile was followed for 20 min after cutting at the specified E/N values. The fragmentation pattern of propanethiol was investigated at the same E/N values by diluting propanethiol into a 10 L tedlar bag (CEL Scientific Corp., Santa Fe Springs, CA, USA) filled with dry zero air.

### 2.4. GC-SCD and GC-MS

A gas chromatograph equipped with a sulfur chemiluminescence detector (GC-SCD, GC 7890 A and SCD 355, Agilent Technologies A/S, Hørsholm, Denmark) was used to measure the compounds emitted 1, 30, 60, 90, and 120 min after cutting. The GC-SCD was equipped with a 1.0 mL sample loop which was continuously flushed with diluted sample air during the experiment. At sampling, the air in the loop was led into the inlet of the GC. The gas chromatograph was equipped with a capillary column with a stationary phase of dimethylpolysiloxane (DB-1, Agilent Technologies A/S). The column had a length of 60 m, an inner diameter of 0.53 mm, and a stationary phase of 5 μm. The helium carrier gas flow rate was set to 10 mL·min^−1^. The GC oven temperature was held for 4 min at 40 °C and ramped to 200 °C at 10 °C·min^−1^. The analysis was performed in triplicates. Propanethiol and dipropyl disulfide was identified using authentic standards diluted into a 10 L tedlar bag (CEL Scientific Corp.) filled with dry zero air. The LOD for GC-SCD is around 1–2 ppb (estimated as 3 times the baseline noise) [27], which is higher than the detection limit of PTR-MS [25–27]. Thermal desorption GC-MS (TD-GC-MS) analysis of onion odor was performed in initial experiments with an instrumentation as described in [26] and compound tentative identification was based on mass spectra matching in the standard NIST-98/Wiley library.

### 2.5. Data Analysis

The data from the continuous PTR-MS analysis were explored by principal component analysis (PCA) using the calculated concentrations in ppb. The PCA analysis is an unsupervised dimension-reducing method enabling visual exploration of correlations and patterns in data [31]. The significant intrinsic variation could be simplified by reducing the complexity of the data sets into two principal components. PCA was calculated using the PLS_Toolbox 6.2 (Eigenvector Research Inc., Wenatchee, WA, USA) in Matlab. In the analysis, pareto scaling [32] was used for pre-processing of the data. The continuous mass spectra were corrected for minor fluctuations in flow rate driven by the instrument by the water cluster signal at *m/z* 37.

## 3. Results and Discussion

### 3.1. Real Time PTR-MS of Freshly Cut Onion Volatiles

Real time PTR-MS was applied to follow the very rapid development of onion odor emitted from freshly cut onions. In general, the VOC emission increased immediately after cutting when the volatiles were enzymatically formed by disruption of the physical barriers between enzymes and precursors.

**Figure 3.**
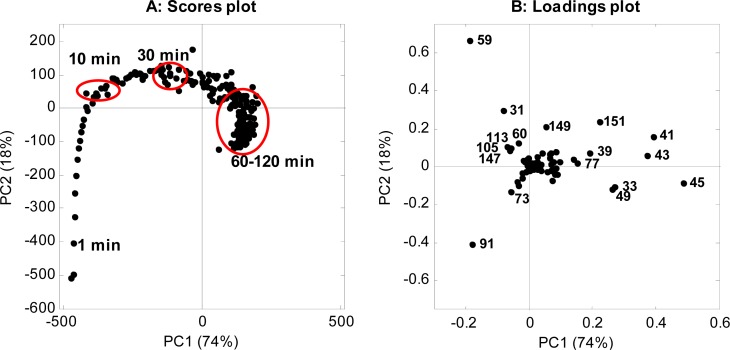
(**A**) Scores plot and (**B**) loadings plot of VOC masses emitted from freshly cut onions during the first 120 min after cutting.

A PCA of the data was performed in order to capture the main variations in the mass spectra over time, and the scores and loadings are shown in Figure 3. The scores plot shows the variation in the mass spectra during the first 120 min after cutting (Figure 3(A)), and each point in the scores plot represents a mass spectrum. The variation can be described as a movement between three time points; (1) the first initial minutes; (2) 10 min after cutting; and (3) a stable period 60–120 min after cutting. During the first initial minutes, the scores were positioned low on PC1 and PC2, and during the first 10 min the scores quickly moved up PC2 and hereafter the scores slowly moved to the right. After 60 min the scores moved slightly down PC2 until the measurement stopped 120 min after cutting. The PCA showed that the VOC composition changed fast during the initial 10 min after cutting, changed some 10–60 min after cutting, and changed less towards the end of the sampling period.

The loadings plot (Figure 3(B)) describes how the different masses contribute to the variation in the scores plot (Figure 3(A)). The masses close to the middle of the loadings plot do not contribute to the variation, while masses positioned further away from the middle do contribute to the variation (Figure 3(B)). The variation along the x-axis, the PC1, mainly explained variation in *m/z* 41, 43, and 45, but also in *m/z* 33, 39, 49, 77, and 151. The variation along the y-axis, the PC2, explains variation in *m/z* 59 and 91, but also in *m/z* 31, 149, and 151. In the first minutes, *m/z* 91 is high, and then *m/z* 31 and 59 rise. The last two masses are placed on the same line from the middle of the loadings plot (Figure 3(B)), indicating that they are co-emitting and that *m/z* 31 could be a fragment of *m/z* 59, or that *m/z* 31 and 59 could be fragments of the same compound. At 60 min, the *m/z* 41, 43 and 45 dominated (Figure 3(B)) and the points hereafter are placed close to each other (Figure 3(A)), which indicate that the volatile profile did not change considerably from 60 to 120 min after cutting.

**Figure 4.**
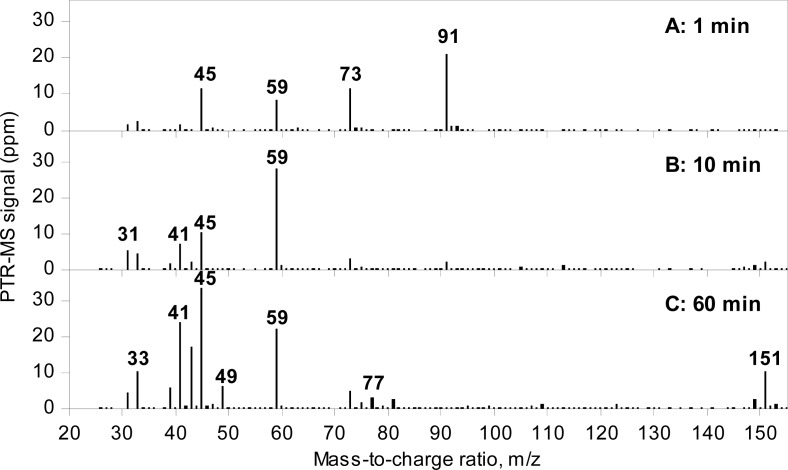
PTR-MS spectra of freshly cut onion volatiles (**A**) 1 min; (**B**) 10 min and (**C**) 60 min after cutting. Data are presented as the averages of three consecutive scans and are corrected for the dilution factor. Water clusters, oxygen (O_2_^+^), and isotopic masses of these are not included.

Full scan mass spectra of the headspace 1, 10 and 60 min after cutting are presented in Figure 4. The composition of the headspace volatiles differed at the three time points, as was expected. One min after cutting, the headspace was dominated by *m/z* 45, 59, 73, and 91 (Figure 4(A)). After 10 min, *m/z* 73 and 91 were almost disappeared, and especially *m/z* 59 was high (Figure 4(B)). After 60 min, many masses were present in the headspace sample e.g., *m/z* 33, 39, 41, 43, 45, 49, 59, 151 (Figure 4(C)).

### 3.2. Identification of VOCs by PTR-MS

In order to assign onion compounds to the masses, a range of reference mass spectra were measured (Table 1). For most of the compounds in Table 1, the protonated molecular ion (MH^+^) is the most abundant ion, and many of the compounds show a high degree of fragmentation. The gas phase ion-molecule reactions happening in the drift tube are thought to consist of several discrete steps [32]. No further try to formulate the complex fragmentation mechanisms was done. 1-Propenyl (isoallyl) compounds were not available, and instead 2-propenyl (allyl) thiosulfinate was synthetized. This compound fragmented considerably (Table 1). It is presumed that the 1-propenyl thiosulfinate will fragment as well. For compounds with no or very little fragmentation of the protonated form, the MH^+^ can be used for quantification. For the remaining compounds, the most abundant *m/z* will normally be used for quantification [32]. However, certain ions e.g., *m/z* 41 and 43 appear as main fragments in many of the compounds in Table 1 and can therefore not be used for quantification of VOCs from freshly cut onions. The same observation with these ions has been done in other complex mixtures of VOCs [34]. Instead the relation between the MH^+^ and the sum of all fragments in each reference mass spectra was used as a calibration factor. It is possible to use a GC-ToF-MS method with a lower LOD than PTR-MS [30], however, the PTR-MS has the advantage of continuous measurements and thereby detection of rapid changes in volatile composition.

### 3.3. Volatile Compounds Detected by GC

In order to supplement the compound identification, GC-SCD measurements were carried out. In the GC-SCD measurement only sulfurous compounds were detected with an equimolar response (according to the number of sulfur atoms in the molecules, the signal from dipropyldisulfide was therefore corrected according to this) and the inlet of the instrument was designed to be inert to sulfurous compounds. The composition and temporal variation of volatile sulfurous compounds (Table 2) agreed very well with the findings from the PTR-MS in Figures 3 and 4. The sampling 1 min after cutting showed one large peak, this was tentatively assigned to the LF, and it did not appear in the chromatogram 30 min after cutting. At this time propanethiol dominated the headspace followed by dipropyl disulfide. Minor amounts of hydrogen sulfide and methanethiol were also detected. The concentration of hydrogen sulfide continued to increase through the experiment, and the content of methanethiol and propanethiol increased until 60 min after cutting and then decreased. The content of dipropyl disulfide peaked at 30 min after cutting and decreased hereafter. Hydrogen sulfide, methanethiol, propanethiol, and dipropyl disulfide are all compounds with low odor threshold values (OTV) (Table 2).

Initial experiments showed that TD-GC-MS analysis of samples collected on adsorbent tubes resulted in large amounts of dipropyl disulfide and only small amounts of propanethiol (data not shown), which has also been seen in other records with similar instrumentation [3,7,8,11–13]. Since both PTR-MS and GC-SCD measurements clearly show a higher concentration of propanethiol, this demonstrates that dipropyl disulfide measured by TD-GC-MS is partly a product of the oxidation and dimerization of propanethiol during sampling and analysis, as has been reported before for methanethiol [36,37]. This emphasizes the importance of being aware of artifact compounds when volatile sulfur compounds are trapped for GC-analysis.

### 3.4. Dynamics in VOC Emission during the First 10 min after Cutting

**Figure 5.**
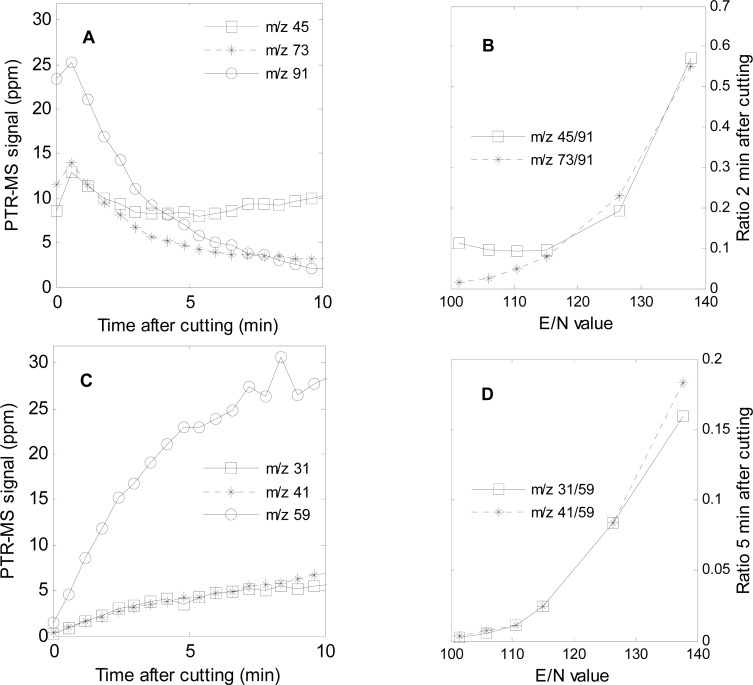
(**A**) Masses associated with the LF emitted 0–10 min after cutting at E/N value 137 Td. (**B**) Ratios *m/z* 45/91 and 73/91 2 min after cutting in experiments at different E/N values. (**C**) Masses associated with propanal emitted 0–10 min after cutting at E/N value 137 Td. (**D**) Ratios *m/z* 31/59 and 41/59 5 min after cutting in experiments at different E/N values.

The mass *m/z* 91 was high from the beginning of the measurement, and during the first few minutes and then decreased (Figures 3(B), 4(A), and 5(A)). Similar trends were seen for *m/z* 45 and 73 (Figures 4(A) and 5(A)). This corresponds to the findings by GC-SCD of a sulfur compound present 1 min after cutting at retention time 10.9 min, which was not present 30 min after cutting (Table 2). Since the LF has a molecular weight of 90 g/mol, the *m/z* 91 and its possible fragments, *m/z* 45 and 73, are tentatively assigned to LF. Propenyl sulfenic acid (Figure 1) could be an alternative as it has the same molecular weight as LF. Considering that other records find the LF in high concentrations in the first minutes after cutting [2,11,14], LF is found to be the most plausible assignment to *m/z* 45, 73 and 91. When *m/z* 91 disappeared after 10 min, *m/z* 45 and 73 continue to appear (Figures 3(B), 4(B) and 5(A)). These ions must therefore also be fragments or MH+ of other compounds than the LF. The ion *m/z* 73 is very likely a fragment of *m/z* 91 as a loss of a neutral water molecule reduces *m/z* 91 to *m/z* 73.

One way of testing if a signal is due to a fragment or a protonated parent compound, is to lower the specific energy input associated with proton transfer (E/N number). At lower E/N, fragmentation will be less pronounced and parent *m/z* signals will be correspondingly higher. Tests of the first 10 min after cutting at different E/N values confirmed that *m/z* 45 and 73 most likely are fragments of *m/z* 91, as the fragmentation ratio decreased at lower E/N values (Figure 5(B)).

No further lowering of the fragmentation ratio *m/z* 45/91 was seen below E/N 115 Td (Figure 5(B)) indicating that some of the signal of *m/z* 45 originated from a MH^+^ and not only a fragment. The identification of fragments in this case is complicated by the temporal changes in abundance of ions (Figure 5(A)). This means that only the signal ratios can be evaluated (Figure 5(B)). However, the systematic variation of signal ratios with E/N is a strong indication that these *m/z* 45 and *m/z* 73 to a large degree are fragments of *m/z* 91. Furthermore, *m/z* 45 continued to increase during the measurement and no other masses co-emitted along with the *m/z* 45 after 10 min. Due to this result, *m/z* 45 remaining after 10 min may very well be assigned to acetaldehyde (Table 2). It is well known that acetaldehyde and ethanol form from anaerobic respiration in response to low oxygen concentrations [39], but in the present study the cut onions were kept at atmospheric air. Consequently, further studies are needed to verify if acetaldehyde is emitted in considerable amounts from freshly cut onions at atmospheric oxygen concentrations.

As the LF disappears, *m/z* 59 increases and reaches a maximum around 10 min after cutting (Figures 3(B), 4(B), and 5(C)). The *m/z* 59 is usually assigned to either acetone or propanal, and the fragmentation pattern with *m/z* 31, 41 and 59 corresponds very well with that of propanal [32] (Table 2). Propanal has been reported as breakdown product of LF in water [1,3]. The fragmentation ratios of *m/z* 31/59 and 41/59 calculated from data 5 min after cutting decreased at lowered E/N value (Figure 5(D)), indicating the m/z 31 and 41 are fragments and *m/z* 59 is a MH^+^. However, *m/z* 41 is also a fragment of other compounds occurring in the headspace of freshly cut onions e.g. propanethiol and dipropyl disulfide (Table 1).

### 3.5. Dynamics in VOC emission 10–120 min after Cutting

**Figure 6.**
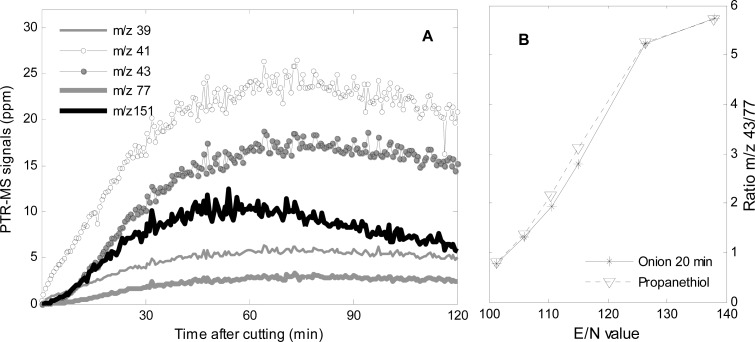
(**A**) Masses associated with propanethiol (*m/z* 39, 41, 43, 77) and dipropyl disulfide (*m/z* 39, 41, 43, 151). Corrected for dilution. (**B**) Ratio *m/z* 43/77 at different E/N values from onion 20 min after cutting and from propanethiol.

Between 10–60 min, *m/z* 39, 41, 43, 77 and 151 appeared in the spectra (Figures 4(C) and 6(A)), and these masses continued to rise until 60 min after cutting and decreased slightly hereafter (Figure 6(A)), however, *m/z* 41 and 43 continued to dominate until the end of the measurement after 120 min.

Propanethiol and dipropyl disulfide can be assigned to respectively *m/z* 77 and 151. Both compounds, and especially propanethiol, fragmented into *m/z* 41 and 43 (Table 1). The fragmentation ratio of *m/z* 43/77 20 min after cutting measured at different E/N values corresponds very well with the fragmentation ratio of propanethiol (Figure 5(B)), which indicate that the majority of *m/z* 43 originates from propanethiol.

**Figure 7.**
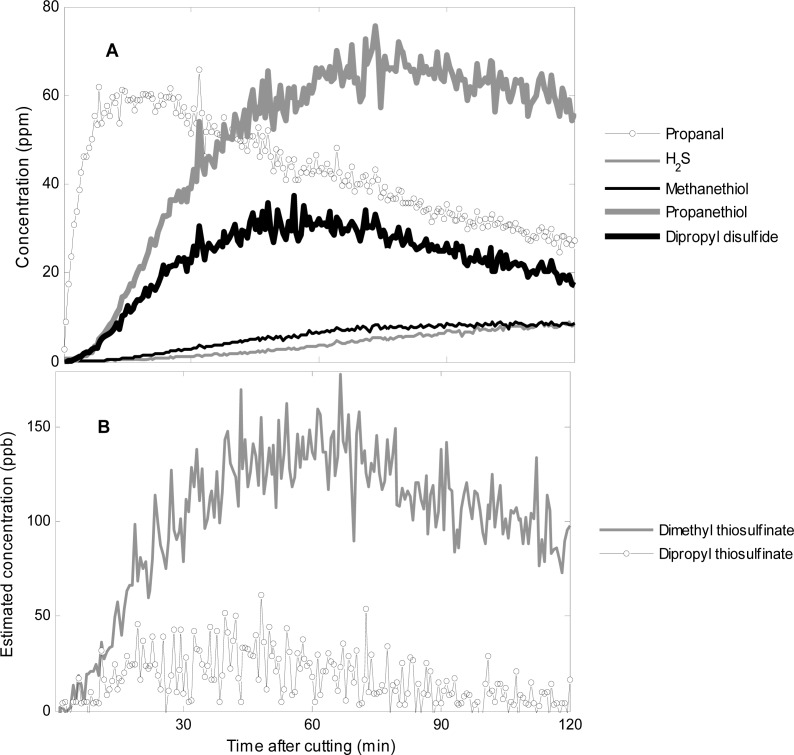
(**A**) Concentration of volatile organic compounds in ppm emitted from freshly cut onions determined by PTR-MS 0–120 min after cutting. The concentration of hydrogen sulfide was corrected for the water content as described in [25] and [26]. (**B**) Concentration in ppb of thiosulfinates emitted from freshly cut onions determined by PTR-MS 0–120 min after cutting. Proton transfer reaction rate constants reported in Table 1 were used for the calculations.

From the fragmentation pattern of propanthiol, dipropyldisulfide, and propanal approximate concentrations were calculated (Figure 7(A)). Using this method of quantification it is clear that propanethiol is the main volatile organic compound emitted from of freshly cut onions 60 min after cutting, which also was the result from the GC-SCD (Table 2). From the sampling of the propanethiol reference compound minor amounts of dipropyl disulfide were obtained (Table 1).

It is possible that some of the measured dipropyl disulfide is a result of dimerization of propanethiol, but the measured concentration of dipropyl disulfide is substantially higher than what would be expected from dimerization of propanethiol during the measurement. Propanal showed a maximum at 10–20 min after cutting. In accordance with the results of the GC-SCD analysis (Table 2), hydrogen sulfide (*m/*z 35, Table 1) and methanethiol (*m/z* 49, Table 1) was found to increase from 10 min after cutting, but at lower concentrations than propanethiol and dipropyl disulfide (Table 2 and Figure 7(A)). Overall, the time of emission of the different compounds and the relation between the compounds was alike in the three repetitions, however, the concentration level varied from experiment to experiment, which can be ascribed to biological variation from onion to onion.

The measurements of pure compounds demonstrated that thiosulfinates can be detected by PTR-MS (Table 1). For onion samples, dimethyl thiosulfinate (*m/z* 111) was detected with a maximum 60 min after cutting (Figure 7(B)), but the concentration was very low (ppb) compared with the other sulfur compounds (ppm) (Figure 7(A)). Trace amounts of other thiosulfinates were detected in the headspace (Figure 6(B)). Likewise, in the GC-SCD analysis no substantial peaks appeared that could be assigned to thiosulfinates.

Given the odor threshold values (OTVs) of the sulfur compounds emitted from freshly cut onions (Table 2) and the relative concentrations of those compounds (Figure 7(A)) odor activity values (OAVs) can be calculated. OAV is a measure of the importance of a specific compound to the odor and is the ratio between the concentration in the sample and the OTV. At 60 min after cutting the OAV of propanethiol is approximately 20 times the OAV of dipropyl disulfide (approximately 28,000 for propanethiol and 1,400 for dipropyldisulfide). This point to that propanethiol is the main compound responsible for onion odor.

Propanethiol from cut onions has only been reported in minor amounts before [7,12] and is therefore not indicated in published schemes of the degradation of sulfoxides [1–3,14]. The timing 30–60 min after cutting and the co-emission with dipropyl disulfide indicate that propanethiol could also result from a reduction of thiosulfinates and sulfenic acids occurring in the onion. Further investigations are needed to determine the pathway of the formation of propanethiol. Thiosulfinates have been associated with “real” onion odor [11], however, the concentration of thiosulfinates was low in our study (Figure 7(B)).

Furthermore, the results indicate that the synthesis of volatile compounds in freshly cut onions is a dynamic process and that important information may be lost by the use of discrete sampling techniques in aroma analysis. These findings suggest that the time from cutting to serving of cut onions is important for sensory perception. It is likely that other vegetables show similar dynamics in the emission of volatiles, as is seen in a recent study of broccoli [23] and based on the current study; PTR-MS will be a very useful tool for investigating such dynamics.

## 4. Conclusions

The investigation by PTR-MS revealed the dynamics of the emission of volatiles and detected the emitted volatile sulfurous compounds and not only the oxidation products created during trapping. By means of PTR-MS, an estimate of the concentrations of different compounds in the headspace of freshly cut onions was achieved. The results demonstrate that PTR-MS is a powerful tool in investigating dynamics in the emissions of VOCs from freshly cut fruits and vegetables. The time from cutting until measurement can have an enormous effect on the results from measuring volatiles and thereby flavor of freshly cut vegetables and this should be taken into account when aroma profiles are investigated.

## Figures and Tables

**Table 1.** Reference mass spectra of compounds used for identification of VOCs from freshly cut onions. Proton transfer reaction rate constants (*k_c_*) used for calculations of relative amounts are listed.

**Reference compounds**	**Mw**	***k_c_* (×10^−9^ cm^3^/s)**	**Fragments *m/z* (Relative intensity)**
Methanol	32	2 **^a^**	33(100 MH^+^)
Hydrogen sulfide	34	- **^b^**	35(100 MH^+^)
Acetaldehyde	44	2 **^a^**	45(100 MH^+^)
Methanethiol	48	2.10 **^c^**	49(100 MH^+^)
Acetone	58	2 **^a^**	59(100 MH^+^)
Propanal	58	2.86 **^c^**	59(100 MH^+^), 31(25), 41(16)
Propanethiol	76	2.16 **^c^**	41(100), 43(60), 39(45), 77(10 MH^+^), 151(1)
Dimethyl disulfide	94	2.45 **^c^**	95(100 MH^+^), 79(32), 97(10), 96(4)
Dimethyl thiosulfinate **^d^**	110	2 **^a^**	111(100 MH^+^), 65(18), 61(17), 43(10), 113(8), 112(4)
Dipropyl disulfide	150	2.87 **^c^**	151(100 MH^+^), 41(37), 43(33), 109(14), 39(9), 152(8), 153(8), 75(4)
Di-2-propenyl disulfide	146	2 **^a^**	73(100), 105(48), 147(39 MH^+^), 45(29), 115(9), 81(8), 75(5), 74(4)
Di-2-propenyl thiosulfinate **^d^**	162	2 **^a^**	41(100), 43(70), 57(45), 39(21)
Dipropyl thiosulfinate **^d^**	166	2 **^a^**	167(100 MH^+^), 41(13), 43(12), 169(10), 168(7), 61(6), 57(5), 73(5)

^a^ Standard proton transfer reaction rate constant.

^b^ Concentration calculated as described in [25,26].

^c^ Proton transfer reaction rate constant calculated after [35] using constants found in NIST.

^d^ Synthesized by the method of Moore and O’Connor [24].

**Table 2.** Volatile compounds found in headspace from freshly cut onions by GC-SCD.

**Compound**	**Minutes after start of cutting (area%)^d^**							
	**RT^a^**	**ID^b^**	**OTV^c^**	**1**	**30**	**60**	**90**	**120**
Hydrogen sulfide	1.76	A	1.9	1	4	10	17	22
Methanethiol	2.65	A	0.07		4	7	8	9
Propanethiol	7.02	A	2.18	1	61	62	57	53
LF	10.90	B	-	97				
Dipropyl disulfide	18.25	A	21.2	1	13	8	7	6

^a^ Retention time in min.

^b^ A, retention time agree with standard; B, tentative assignment according to comparison of time after cutting with literature review.

^c^ Odor threshold values (OTV) (ppb) were based on reported detection threshold values [38].

^d^ Average of three replicates.
